# Fibroblast-Derived Extracellular Matrix Induces Chondrogenic Differentiation in Human Adipose-Derived Mesenchymal Stromal/Stem Cells in Vitro

**DOI:** 10.3390/ijms17081259

**Published:** 2016-08-03

**Authors:** Kevin Dzobo, Taegyn Turnley, Andrew Wishart, Arielle Rowe, Karlien Kallmeyer, Fiona A. van Vollenstee, Nicholas E. Thomford, Collet Dandara, Denis Chopera, Michael S. Pepper, M. Iqbal Parker

**Affiliations:** 1 International Centre for Genetic Engineering and Biotechnology (ICGEB), Cape Town Component, Anzio Road, Observatory, Cape Town 7925, South Africa; taegyn.turnley@alumni.uct.ac.za (T.T.); andrew.wishart@alumni.uct.ac.za (A.W.); arielle.rowe@icgeb.org (A.R.); iqbal.parker@icgeb.org (M.I.P.); 2 Division of Medical Biochemistry, Institute of Infectious Disease and Molecular Medicine, Faculty of Health Sciences, University of Cape Town, Anzio Road, Observatory, Cape Town 7925, South Africa; 3 Department of Immunology, Institute for Cellular and Molecular Medicine, South African Medical Research Council (SAMRC) Extramural Unit for Stem Cell Research and Therapy, Faculty of Health Sciences, University of Pretoria, Pretoria 0002, South Africa; karlienkallmeyer@gmail.com (K.K.); fionavanvollenstee@gmail.com (F.A.v.V.); michael.pepper@up.ac.za (M.S.P.); 4 Division of Human Genetics, Department of Pathology, Institute of Infectious Diseases and Molecular Medicine, Faculty of Health Sciences, University of Cape Town, Anzio Road, Observatory, Cape Town 7925, South Africa; thmnic023@myuct.ac.za (N.E.T.); collet.dandara@uct.ac.za (C.D.); 5 Division of Immunology, Faculty of Health Sciences, University of Cape Town, Anzio Road, Observatory, Cape Town 7925, South Africa; denis.chopera@gmail.com

**Keywords:** mesenchymal stromal/stem cells, regenerative medicine, three-dimensional, extracellular matrix, differentiation, chondrogenesis

## Abstract

Mesenchymal stromal/stem cells (MSCs) represent an area being intensively researched for tissue engineering and regenerative medicine applications. MSCs may provide the opportunity to treat diseases and injuries that currently have limited therapeutic options, as well as enhance present strategies for tissue repair. The cellular environment has a significant role in cellular development and differentiation through cell–matrix interactions. The aim of this study was to investigate the behavior of adipose-derived MSCs (ad-MSCs) in the context of a cell-derived matrix so as to model the in vivo physiological microenvironment. The fibroblast-derived extracellular matrix (fd-ECM) did not affect ad-MSC morphology, but reduced ad-MSC proliferation. Ad-MSCs cultured on fd-ECM displayed decreased expression of integrins α2 and β1 and subsequently lost their multipotency over time, as shown by the decrease in CD44, Octamer-binding transcription factor 4 (*OCT4*), *SOX2*, and *NANOG* gene expression. The fd-ECM induced chondrogenic differentiation in ad-MSCs compared to control ad-MSCs. Loss of function studies, through the use of siRNA and a mutant Notch1 construct, revealed that ECM-mediated ad-MSCs chondrogenesis requires Notch1 and β-catenin signaling. The fd-ECM also showed anti-senescence effects on ad-MSCs. The fd-ECM is a promising approach for inducing chondrogenesis in ad-MSCs and chondrogenic differentiated ad-MSCs could be used in stem cell therapy procedures.

## 1. Introduction

The extracellular matrix (ECM) is a complex network of a variety of components that interact to create a molecular scaffold [1,2]. The ECM is secreted by tissue-specific cells that are found mostly in connective tissue and is able to support cells within its reach [2,3]. It acts as a substrate for cell attachment and migration, while presenting physical and chemical cues to cells. ECM molecules include proteoglycans, collagens, glycosaminoglycans, and non-collagenous glycoproteins. Cells continue to interact with the ECM they produce and that produced by other cells. The composition of the ECM is in a constant flux and varies depending on factors such as cell and tissue type. The mechanical and biochemical properties of the ECM are the two key factors influencing cellular behavior [4,5,6]. The extracellular matrices (ECMs) of different tissues show differences that provide specific signaling for cells of that tissue. ECMs of specific tissues induce cell proliferation that is specific for that tissue and is involved in maintenance of that cell phenotype [7]. The ECM regulates the function of cells and drives cellular differentiation. Thus ECMs show bioactivities including inducing proliferation and differentiation [2,5,8,9]. The in vitro bioactivities of many synthetic and natural ECMs are not fully understood. Our previous work has demonstrated that ECMs play a role in the specification of cell fate, especially that of stem cells [10,11].

One of the greatest challenges in utilizing stem cells for tissue repair is directing gene regulation and guiding stem cell differentiation toward a specific lineage [1,4,9,10,12,13,14]. Mesenchymal stromal/stem cells (MSCs) have been shown to be able to proliferate considerably and can differentiate into various tissue lineages when induced. For the use of MSCs in regenerative or reparative therapeutic processes to become a reality, an understanding of the processes and signaling pathways that effectively induce MSCs differentiation along key lineages is imperative. Several studies have been able to exhibit this with varying degrees of success [9,10,13,14]. However, the lack of consistency and the inability to replicate the same results with repeat experiments is still a challenge. The current methods used for MSCs expansion in vitro limit the use of MSCs due to many reasons including impaired multi-lineage differentiation during monolayer culture [15]. Thus research aimed at directing MSC differentiation along certain lineages is ongoing. Recent work on the use of matrices to drive stem cell differentiation has focused on the use of combinatorial matrices and hydrogels [16,17,18,19]. There is a gradual realization that three-dimensional ECM (3D ECM) models better recapitulate the in vivo situation, as cells reside and differentiate within a 3D microenvironment in the body [4,5]. The use of 3D ECM models in tissue engineering studies will also provide more accurate data or outcomes regarding the relevance and potential use of the information in clinical applications. The incorporation of MSCs into synthetic biomaterial scaffolds such as hydrogels and polymers such as polyglycolic acid (PGA) are some of the techniques being used to expand and differentiate MSCs into the desired cells in vitro [16,17,18,19]. However, these synthetic polymer-based scaffolds lack biocompatibility and are not suitable when cells are intended to be used therapeutically. Therefore, more ECMs and their combinations are being researched in order to understand their effect on MSC proliferation and differentiation.

Recent studies have shown that cell-derived extracellular matrices (cd-ECMs) can promote the proliferation of cells such as fibroblasts and MSCs, and are able to maintain a high responsiveness in vitro [4,5,20]. In vivo, MSCs are found within microenvironments where the ECM is also synthesized by cells such as fibroblasts. In particular, ad-MSCs are found in close proximity to several cell types collectively termed the stromal vascular fraction, which is rich in stromal cells such as preadipocytes, fibroblasts, and macrophages; therefore, the ECM will mostly be of stromal origin [7,21]. It is therefore reasonable to study how a fibroblast-derived ECM will affect ad-MSCs in vitro instead of using synthetic ECMs. In this study, therefore, we used a fibroblast-derived extracellular matrix (fd-ECM) to evaluate its effect on ad-MSCs, focusing specifically on ad-MSC proliferation, attachment, migration, and possible differentiation. We also determined the signaling pathways perturbed by the fd-ECM. We show in this study that both Notch1 and β-catenin signaling were activated by the presence of the fd-ECM and this led to chondrogenic differentiation of ad-MSCs. The fd-ECM also has an anti-senescence effect and may improve the survival of ad-MSCs in culture.

## 2. Results

### 2.1. Adipose-Derived Mesenchymal Stromal/Stem Cells (MSCs) Characterization

**Figure 1:**
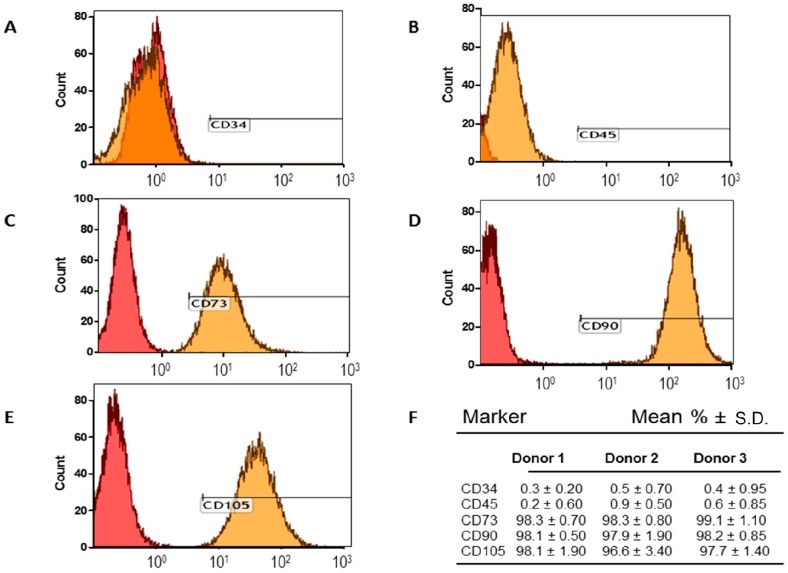
The phenotypic characterization of adipose-derived mesenchymal stromal/stem cells (ad-MSCs). (**A**–**E**) Flow cytometric analysis of ad-MSCs at passage 6–10 was performed as described in the “Materials and Methods” Section 4.9; (**F**) Average of cells (%) staining positive for MSC surface epitopes as described above. The classical MSC phenotype is when the cells have the phenotype CD73^+^, CD90^+^, CD105^+^, CD45^−^ and CD34^−^ in ≥95% of the cell population. Different ad-MSC preparations were used during the characterization; for the sake of brevity we show representative results for three donors. Mean% ± S.D. (standard deviation).

**Figure 2:**
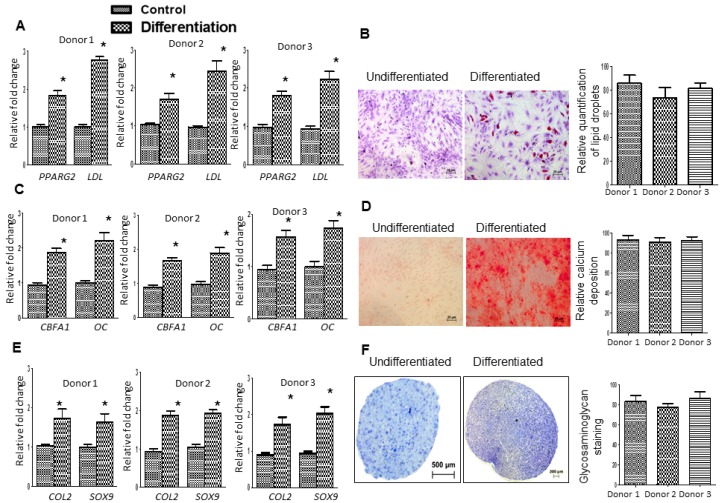
Lineage-specific differentiation capacity of ad-MSCs. (**A**,**B**) RT-qPCR was done to evaluate adipogenesis markers, *Peroxisome proliferator-activated receptor γ 2 (PPARG2*), and *Lipoprotein lipase (LPL*). Quantification of lipid droplets is shown in (**B**) after staining undifferentiated and differentiated samples with Oil Red O. Scale bar: 20 µm; (**C**,**D**) RT-qPCR was done to evaluate osteogenesis markers, *Core-binding factor subunit α1 (CBFA1*) and *Osteocalcin (OC*). Undifferentiated and differentiated donor ad-MSCs were stained with Alizarin red S. In osteogenic cultures mineralization was visible as red-stained calcium deposition in (**D**). Scale bar: 20 µm; and (**E**,**F**) RT-qPCR was done to evaluate chondrogenesis markers such as type II collagen (*COL2*) and *Sox9*. Undifferentiated and differentiated donor ad-MSCs were stained with Toluidine blue O. In chondrogenic cultures staining of the proteoglycans (purple) was visible. Quantification of glycosaminoglycans production is shown. Scale bar: Undifferentiated: 500 µm; Differentiated: 200 µm. * *p* < 0.05.

**Figure 3:**
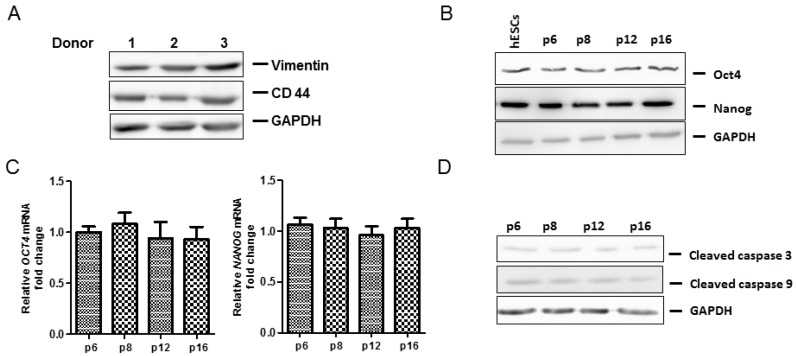

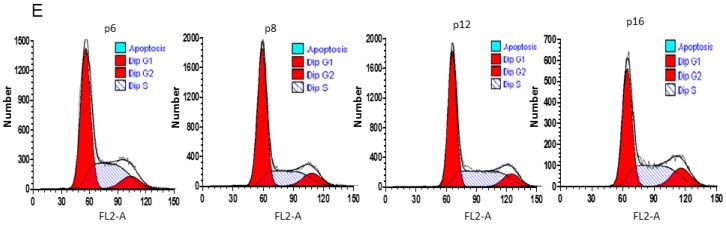
Ad-MSCs express pluripotency markers and are viable over several passages. (**A**) Vimentin and CD44 expression in ad-MSCs from three donors was determined by immunoblot analysis. GAPDH was used as a loading control; (**B**,**C**) Ad-MSCs express Octamer-binding transcription factor 4 (Oct4) over several passages. The expression of pluripotency markers Oct4 and Nanog expression in ad-MSCs was determined over several passages was determined by immunoblot analysis and RT-qPCR; (**D**) Cleaved caspase 3 and 9 in ad-MSCs was determined by immunoblot analysis over several passages; and (**E**) Flow cytometric analysis was done to determine the effect of prolonged culture on ad-MSCs’ cell cycling.

Ad-MSCs were characterized by identification of cell surface markers via flow cytometric analysis. Ad-MSCs expressed CD73, CD90, and CD 105, and did not express CD34 and CD45 (Figure 1A–F). Classic phenotypic characterization of MSCs includes the expression of CD73^+^, CD90^+^, CD105^+^, CD34^−^ and CD45^−^ in ≥95% of the cell population (Figure 1F). Ad-MSCs were able to differentiate into multiple lineages (adipogenic, osteogenic, and chondrogenic) after cultivation in the respective differentiation media for 21 days (Figure 2A–F). Staining was done to show lipid vacuoles, calcium deposits, and glycosaminoglycans. Ad-MSCs were further characterized by evaluating the expression of several mesenchymal stromal/stem and self-renewal markers, such as vimentin, CD44, octamer-binding transcription factor 4 (Oct4), and Nanog via immunoblot analysis (Figure 3A–C). Ad-MSCs from three donors were found to express the MSC marker vimentin and CD44. Our data also show that ad-MSCs can differentiate into mesodermal cell lineage (Wnt Family Member 3a: *WNT3A*, *N-CADHERIN* mRNA), ectodermal differentiation (*CALBINDIN2* and *RECOVERIN* mRNA), and endodermal differentiation (*PANCREATIC AMYLASE* mRNA, *SOX17* mRNA) (Figure S1A–F). Sub-culturing the ad-MSCs did not affect the expression of Oct4 or Nanog, showing that ad-MSCs maintain their multipotency and there is no induction of apoptosis over several passages (Figure 3B–D). Flow cytometric analysis showed that, over several passages, the ad-MSCs maintained the same cell cycle pattern (Figure 3E).

### 2.2. Proliferative Capacity and Morphology of Adipose-Derived MSCs (Ad-MSCs) Cultured on a Fibroblast-Derived Extracellular Matrix (Fd-ECM)

**Figure 4:**
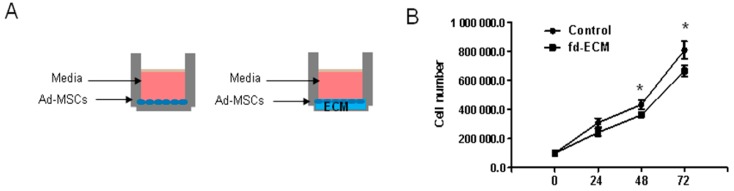

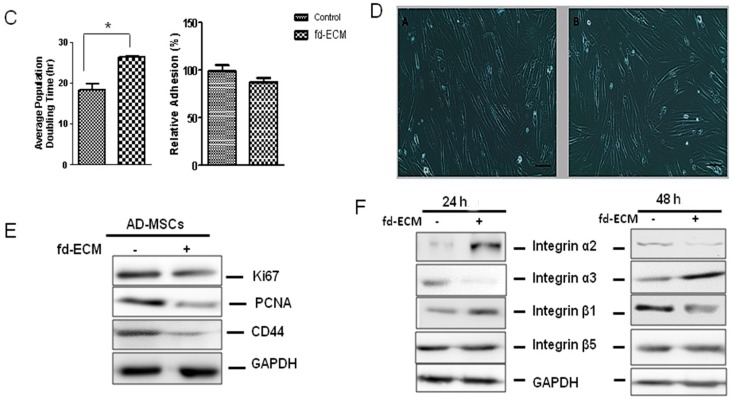
Fibroblast-derived extracellular matrix (Fd-ECM) reduces ad-MSCs proliferation. (**A**) Schematic representation of the experimental setup. Ad-MSCs were cultured on control plastic dishes (−) and on dishes containing fd-ECM (+); (**B**) Ad-MSCs were cultured on control dishes (−) and on the fd-ECM (+) for the indicated time periods. Control ad-MSCs (−) and those plated on fd-ECM (+) were counted at the indicated times using the Countess Cell Counter; (**C**) Average ad-MSCs population doubling time was calculated using the method described in “Materials and Methods Section 4.7”. Relative adhesion of ad-MSCs cultured on plastic dishes and on fd-ECM was evaluated and expressed as a percent of control cells. Each experiment was performed in triplicate; (**D**) Ad-MSCs were cultured on control dishes and on the fd-ECM for 48 h and cell images were taken using an Olympus CKX41 microscope. Scale bar: 25 µm; (**E**) The expression of Ki67, proliferating cell nuclear antigen (PCNA), and CD44 decreased significantly in ad-MSCs cultured on fd-ECM (+) as determined by immunoblot analysis and (**F**) Immunoblot analysis of integrin α2, α3, β1, and β5 in ad-MSCs lysates after culture on plastic (−) and on fd-ECM (+) for 24 and 48 h. * *p* < 0.05.

Ad-MSC proliferative capacity was analyzed by determining the growth kinetics of MSCs by direct cell counting (Figure 4A). Cell numbers at each time point indicated significant differences in proliferation between MSCs grown on control plastic dishes and those cultured on fd-ECM (Figure 4B). Cell growth rate was also determined by evaluating the population doubling time during successive subcultures. Indeed, the average population doubling time for ad-MSCs on the fd-ECM increased significantly (Figure 4C). There was no significant change in cellular adhesion to the fd-ECM compared to the control dishes (Figure 4C). The morphological differences between MSCs plated on plastic (control) dishes and those plated on fd-ECM (matrix) were observed by phase-contrast microscopy. Images were captured at the end of the 48-h time point at 100× magnification and no major changes in morphology were observed between MSCs grown on control dishes and those cultured on fd-ECM (Figure 4D). The expression of proliferative markers, proliferating cell nuclear antigen (PCNA), and Ki67 were analyzed by immunoblot analysis and ad-MSCs cultured on the fd-ECM show significant decrease in Ki67, PCNA, and CD44 protein levels after 48 h of incubation (Figure 4E). After 24 h of culture on the fd-ECM significant increases were observed in integrins α2 and β1 while integrin α3 was downregulated (Figure 4F). Significant decreases in integrins α2 and β1 were observed in ad-MSCs cultured on fd-ECM compared to controls after 48 h of culture on the fd-ECM (Figure 4F).

**Figure 5:**
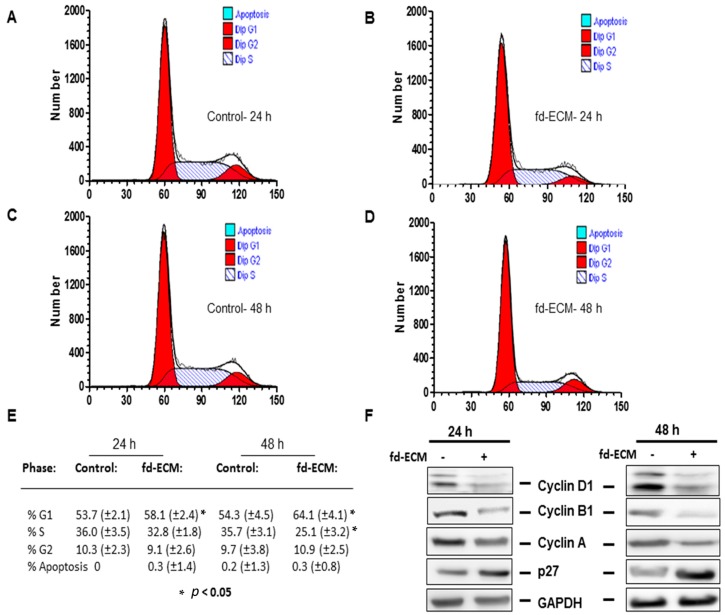
Fd-ECM downregulates cell cycling in ad-MSCs in vitro. Ad-MSCs were cultured on plastic dishes (−) or on fd-ECM (+) for the indicated time periods (**A**) Flow cytometric analysis of control ad-MSCs showed no apparent apoptosis after 24 h of incubation; (**B**) Ad-MSCs were cultured on fd-ECM for 24 h and cell cycle analysis was done by flow cytometry after labeling ad-MSCs with propidium iodide; (**C**) Ad-MSCs were cultured on tissue plastic dishes for 48 h, labeled with propidium iodide for flow cytometric analysis; (**D**) Ad-MSCs were cultured on fd-ECM for 48 h and flow cytometric analysis was done as described above; (**E**) Ad-MSCs (%) in each cell cycle stage after ad-MSCs were cultured on plastic and on fd-ECM for 24 and 48 h. Four different experiments were pooled. Results show mean ± standard deviation; and (**F**) Fd-ECM reduces cycling in ad-MSCs. Immunoblot analysis of cyclin D1, cyclin B1, cyclin A, and p27 in ad-MSCs lysates after culture on plastic (−) and fd-ECM (+) for 24 and 48 h.

In addition, a semi-quantitative assessment of whether the fd-ECM affects ad-MSCs’ cell cycle progression was made. Cell cycle analysis was done by flow cytometry on ad-MSCs plated on plastic (control) and fd-ECM after propidium iodide labeling. Significant differences in both the G1 and S phases were observed between control cells and those plated on fd-ECM. After 24 h of incubation there appear to be a G1 cell-cycle arrest when cells were cultured on fd-ECM (Figure 5A,B,E). Approximately 10% of the ad-MSCs were detected in the G2 phase of the cell cycle. After 48 h of incubation, there was also a significant increase in ad-MSCs in the G1 phase while ad-MSCs in the S phase decreased significantly (Figure 5C–E). Immunoblot analysis showed a downregulation of cyclin D1, cyclin B1, and cyclin A, and an increase in p27 (Figure 5F). This downregulation, coupled with the decrease in Ki67 expression, is not associated with actively cycling ad-MSCs but with those undergoing differentiation. These results suggest that the fd-ECM does not promote ad-MSC proliferation but induces differentiation of ad-MSCs.

### 2.3. Fd-ECM Directs Ad-MSCs Differentiation towards the Chondrogenic Lineage

**Figure 6:**
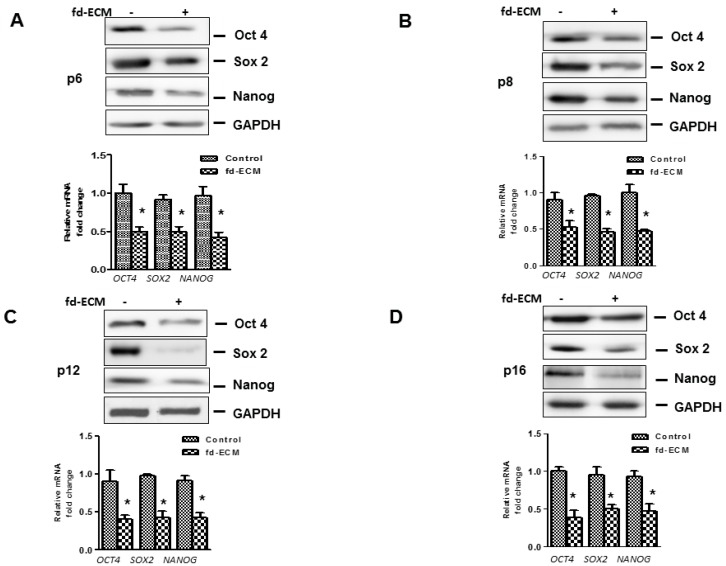
Fd-ECM downregulates pluripotency genes expression over several passages. Ad-MSCs were cultured on plastic dishes (−) or on fd-ECM (+) for the indicated passages (**A**) Protein and mRNA levels of Oct4, Sox2, and Nanog were determined in ad-MSCs lysates and mRNA at passage 6; (**B**) Protein and mRNA levels of *OCT4*, *SOX2*, and *NANOG* were evaluated in ad-MSCs lysates and mRNA at passage 8; (**C**) Protein and mRNA levels of *OCT4*, *SOX2*, and *NANOG* were evaluated in ad-MSCs lysates and mRNA at passage 12; and (**D**) Protein and mRNA levels of *OCT4*, *SOX2*, and *NANOG* were determined in ad-MSCs lysates and mRNA at passage 16. * *p* < 0.05.

**Figure 7:**
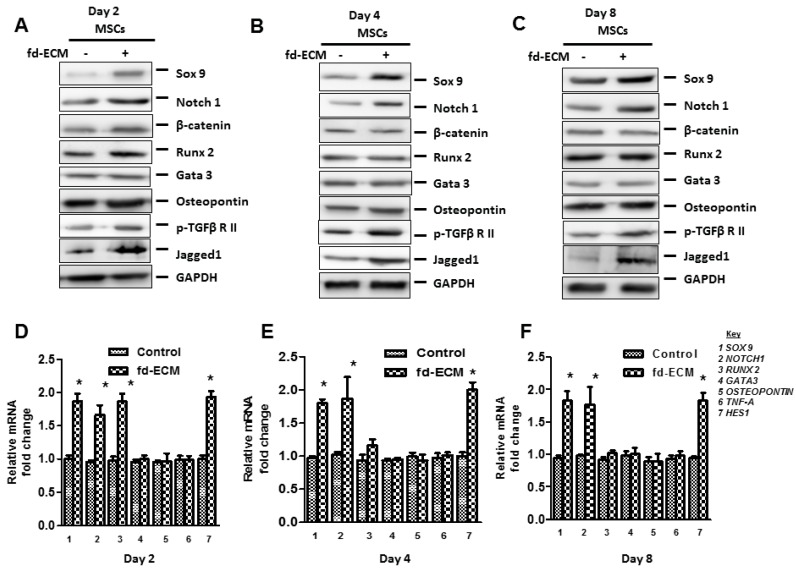
Fd-ECM induces chondrogenic differentiation in ad-MSCs. Ad-MSCs were cultured on plastic dishes (−) or on the fd-ECM (+) for the indicated days (**A**–**C**) Immunoblot analysis of Sox9, Notch1, β-catenin, Runx2, Gata3, Osteopontin, *p*-TGFβRII, and Jagged1 in ad-MSCs lysates after two, four, and eight days of incubation on plastic dishes and on the matrix; (**D**–**F**) RT-qPCR was used to analyze the levels of *SOX9*, *NOTCH1*, *RUNX2*, *GATA3*, *OSTEOPONTIN*, *TNF-α*, and *HES1* in ad-MSCs mRNA after two, four, and eight days of incubation on plastic dishes and on fd-ECM. * *p* < 0.05.

**Figure 8:**
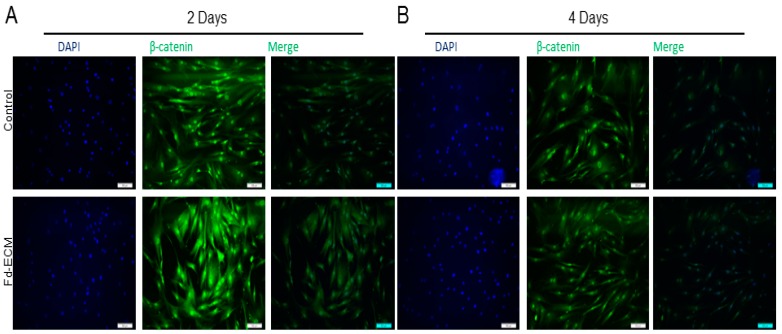

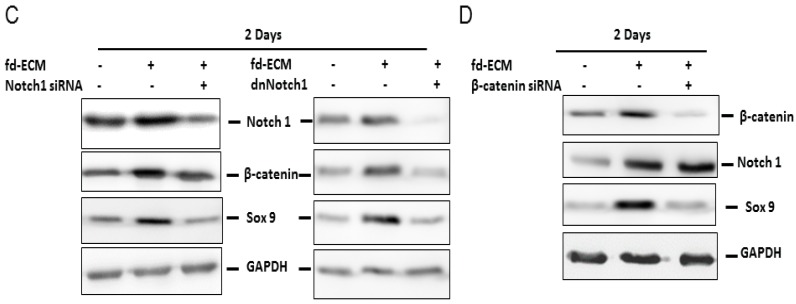
Chondrogenic differentiation of ad-MSCs in the presence of fd-ECM requires activation of Notch1 and β-catenin signaling. Ad-MSCs were cultured on plastic dishes (−) or on the fd-ECM (+) for the indicated number of days (**A**,**B**) Ad-MSCs cultured on plastic control dishes and on fd-ECM were evaluated for β-catenin expression using immunofluorescence assay. Ad-MSCs, cultured for two days and four days, were incubated with antibodies against β-catenin and DAPI was used to stain the DNA in the nucleus. Scale bar: 100 µm; (**C**) Ad-MSCs were transfected with Notch1 siRNA and the dominant negative Notch1 construct using Transfectin Lipid reagent. Evaluation of Notch1, Sox9 and β-catenin protein levels was done; and (**D**) ad-MSCs were transfected with β-catenin siRNA as described above and Notch1, Sox9, and β-catenin protein level was determined.

In order to explore the influence of the fd-ECM on ad-MSCs differentiation, we first analyzed the influence of the matrix on multipotency-associated endogenous gene expression in ad-MSCs over time. Immunoblot analysis and RT-qPCR analysis showed that ad-MSCs cultured on fd-ECM, from passage 6 to passage 16, displayed decreased Oct4, Sox2, and Nanog protein levels (Figure 6A–D). Further analysis to evaluate adipogenic, osteogenic, and chondrogenic differentiation of MSCs cultured on plastic and fd-ECM over the first eight days of culture was done. Differentiation was evaluated using RT-qPCR and immunoblot analysis on differentiation markers. On day 2 after culture, ad-MSCs seeded on fd-ECM showed significantly higher expression levels of Sox9, Notch1, β-catenin, Runx2, *p*-TGFβRII, and Jagged1 compared to controls (Figure 7A). We further assayed the expression of the same genes after four and eight days of incubation. Our results show that Sox9, Notch1, *p*-TGFβRII, and Jagged1 are upregulated in ad-MSCs cultured on fd-ECM after four and eight days of incubation (Figure 7B–F; Figure S2A–D). β-Catenin was significantly upregulated within the first two days of culture on the fd-ECM and thereafter the expression levels were similar to controls (Figure 8A,B; Figure S2A,C,D). Chondrogenic differentiation was also estimated by evaluating the expression of type I collagen and type II collagen. Our results show a gradual decrease in COL1A1 and integrin α2 protein levels and a gradual increase in COL2 synthesized by the ad-MSCs cultured on the fd-ECM (Figure S2C). Increase in the expression of Sox9, Notch1, Jagged1, COL2, and *p*-TGFβRII protein levels is associated with chondrogenic differentiation. This indicates that ad-MSCs seeded on fd-ECM were differentiating towards chondrogenic cell types compared to those on plastic. Our immunofluorescence data substantiated the above results, showing that β-catenin only increased on day 2 and decreased thereafter, whereas Sox9, Notch1, Jagged1, and *p*-TGFβRII increased from day 2 onwards in the presence of fd-ECM (Figure 8A,B; Figure S2A–D). Loss of function studies using Notch1 and β-catenin siRNA and a dominant negative Notch1 construct showed that both β-catenin and Notch1 are important in fd-ECM-mediated ad-MSC differentiation towards the chondrogenic lineage (Figure 8C,D; Figure S3A–D). However, it appears that β-catenin is required during the early stages of differentiation only and that during the later stages of differentiation only Sox9, Notch1, *p*-TGFβRII, and Jagged1 are important.

### 2.4. Anti-Senescence Effect of Fd-ECM on Ad-MSCs

**Figure 9:**
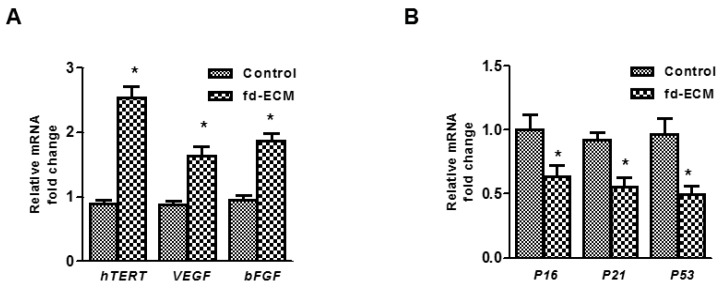

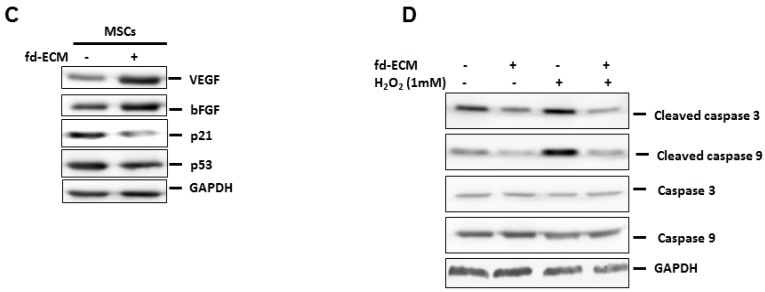
Anti-senescence effect of fd-ECM on ad-MSCs. (**A**) Ad-MSCs were cultured on plastic dishes (−) and on fd-ECM (+) for 48 h, harvested, and total RNA extracted. RT-qPCR was performed to evaluate human Telomerase Reverse Transcriptase (*hTERT*), *VEGF*, and *bFGF* mRNA levels; (**B**) Ad-MSCs were cultured on plastic and on an fd-ECM for 48 h, harvested, and total RNA extracted. RT-qPCR was performed to evaluate *P16*, *P21*, and *P53* mRNA levels; (**C**) Ad-MSCs were cultured on plastic (−) and on fd-ECM (+), harvested, and immunoblot analysis was performed to evaluate VEGF, bFGF, p21, and p53 protein levels; and (**D**) ad-MSCs were cultured on plastic (−) and on an fd-ECM (+) and immunoblot analysis was performed to evaluate cleaved caspases 3 and 9 protein levels. * *p* < 0.05.

Cellular senescence is known to occur during the long-term culture of cells. Thus for ad-MSCs to be used in cell therapy there is a need to manage epigenetic modifications during clonal expansion. We evaluated the possible effect of fd-ECM on ad-MSC senescence. Our results suggest that there is no senescence occurring during the culture of ad-MSCs on fd-ECM. Several genes associated with senescence and anti-senescence were evaluated by RT-qPCR. Gene expression of several anti-senescence markers was significantly upregulated in ad-MSCs cultured on fd-ECM (Figure 9A,C). The expression of several genes such as *P16*, *P21*, and *P53* was downregulated in the presence of fd-ECM (Figure 9B,C; Figure S4A–D). We then evaluated the cell-protective effect of fd-ECM on ad-MSCs. Ad-MSCs plated on the fd-ECM and control cells were exposed to H_2_O_2_ for an hour and a half and then the viability of the ad-MSCs was determined. Ad-MSCs cultured on fd-ECM were more viable compared to ad-MSCs exposed to H_2_O_2_. Immunoblot analysis substantiated these results as the increase in cleaved caspases 3 and 9 observed in the presence of H_2_O_2_ was slightly reduced in the presence of fd-ECM (Figure 9D; Figure S5A,B). Thus fd-ECM appears to reduce the effect of oxidizing agents on ad-MSCs.

### 2.5. Effect of Fd-ECM on Transformation Markers

**Figure 10:**
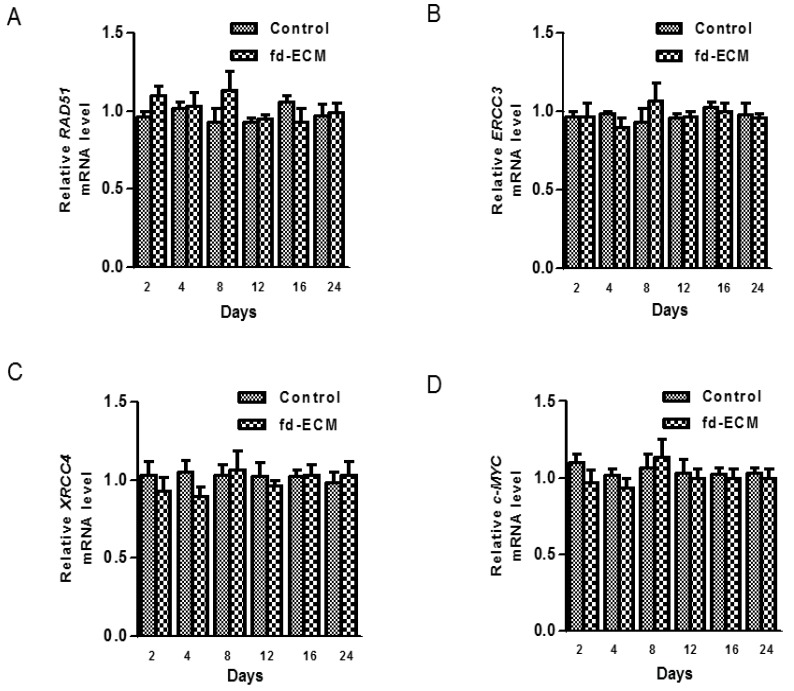
Fd-ECM does not affect transformation markers’ gene expression. (**A**) Ad-MSCs were cultured on plastic and on an fd-ECM for the indicated periods, harvested, and total RNA extracted. RT-qPCR was performed to evaluate *RAD51* mRNA levels; (**B**) *ERCC3* mRNA levels; and (**C**) *XRCC4* mRNA levels; and (**D**) *c-MYC* mRNA levels.

Since ad-MSCs are to be used for various therapeutic procedures, their biosafety needs to be evaluated. Therefore we analyzed the expression of several genes such as *RAD51*, *ERCC3*, *XRCC4*, and *c-MYC* in MSCs cultured on fd-ECM. For fd-ECM to be considered for further studies on tissue engineering applications, the transformation capacity of MSCs cultured on fd-ECM had to be evaluated. The mRNA levels of the transformation markers, *ERCC3*, *RAD51*, *c-MYC*, and *XRCC4* were analyzed using RT-qPCR and the fd-ECM had no significant effect on the mRNA levels of the transformation markers (Figure 10A–D).

## 3. Discussion

The differentiation potential and immunomodulatory properties of MSCs has attracted attention regarding their application in cellular therapies for many pathological conditions [22,23,24,25,26]. MSCs are now obtained from different tissues of the body, making it possible to use MSCs from a given patient in an autologous manner [27,28,29,30,31]. Besides differentiating into endodermal and mesodermal (osteocytes and chondrocytes) lineage cells, MSCs also have remarkable translineage differentiation capabilities into neuronal, retinal, and pancreatic β cells, thereby making them very attractive for regenerative medicine [25,31]. The stem cell niche has been documented to play a significant part in determining the fate of many cells including stem cells and the ECM is also a constituent of this niche [4,10]. In vivo, MSCs are found in environments in which other cells such as fibroblasts predominate [22,23,24,25,26]. In particular, ad-MSCs are found in close proximity to several cell types collectively termed the stromal vascular fraction; this environment is rich in stromal cells such as preadipocytes, fibroblasts, and macrophages and therefore the ECM will mostly be of stromal origin [7,21,32,33,34,35]. In this study, therefore, we determined the influence of fd-derived ECM on ad-MSCs. Cell-derived ECMs from cells such as fibroblasts recapitulate the microenvironments in which MSCs reside and function. Our studies and those of others have shown the importance of different ECMs in determining the fate of many cells including stem cells during differentiation [9,10,36,37]. However, the influence of the 3-D fd-ECM on ad-MSCs has not been studied. In order for ad-MSCs to be used in stem cell therapy, understanding the molecular processes involved in the differentiation of such cells is vital. Ad-MSCs can be obtained from the adipose tissue in abundant amounts and have proven multi-lineage differentiation abilities [23,24,26]. A large amount of adipose tissue is discarded as medical waste and therefore adipose-derived MSCs can be obtained in large quantities with minimal invasive procedures and can be cultured in vitro [32,38].

Of significance, our microscopic and flow cytometric analysis of ad-MSCs shows that ad-MSCs are not affected by subconfluent passaging. Subconfluent passaging resulted in ad-MSCs maintaining their MSC properties for several passages. Our data also show that when MSCs are subconfluent passaged they do not compromise their morphological features. They were also shown to maintain their multipotency and MSC properties such as expression of cell surface markers and lineage differentiation. Many studies have shown that MSCs express Oct4, Nanog, and CD44 [9,11,39,40]. Our results are in agreement with these studies as we observed the expression of stemness markers Oct4 and Nanog. The expression of proteins such as Oct4 is known to be affected by passaging over time [41,42]. The expression of these two stemness markers was maintained at least up to passage 22 in this study and the ad-MSCs had the usual spindle shape typical of fibroblasts. Beyond passage 22, we did not assess the characteristic profile of these ad-MSCs.

This study showed that the fd-ECM reduces ad-MSC proliferation without compromising their morphological features. Cyclin D1 is known to be involved in the G1 and S phases of cell cycle. Our study shows that ad-MSCs cultured on the fd-ECM exhibited decreased expression of cyclin D1 and this might account for the reduced proliferation observed. The reduction in ad-MSC proliferation is coupled with a reduction in stemness and self-renewal gene expression. We further show that the reduction in stemness and self-renewal marker gene expression occurs at the same time as the increase in chondrogenic markers, such as Sox9, Notch1, type II collagen, *p*-TGFβRII, and β-catenin. There is also a slight increase in Runx2 protein levels on day 2 of incubation on the fd-ECM. Our results also show a change in the type of collagen synthesized by the ad-MSCs from type I collagen to type II collagen. This occurs at the same time as a change in integrin synthesis from integrin α2 and β1 to integrin α3. The differentiation of the ad-MSCs is in line with an increase in ad-MSCs in the G1 phase and reduced amounts of ad-MSCs in the S phase. This data illustrates that the fd-ECM induces chondrogenic differentiation of ad-MSCs compared to those on control plastic dishes.

It has been reported that culture of MSCs affects their osteogenic and chondrogenic differentiation [36,39,40,43]. MSCs, like most cells, have been shown to be responsive to their environments, adapting their function and phenotype to the surrounding circumstances [11,32,39]. This study shows that the fd-ECM induces chondrogenic differentiation of ad-MSCs in vitro. Several lineage markers, such as osteopontin, Gata3, Runx2, β-catenin, Sox9, and Notch1, were analyzed using RT-qPCR and immunoblotting. We observed that Sox9, Notch1, Jagged1, β-catenin, and Runx2 were upregulated 48 h after culture of ad-MSCs on the ECM. Longer incubation of ad-MSCs on the ECM caused an upregulation of Sox9, *p*-TGFβRII, Notch1, Notch1 ligand, Jagged1, and Hes1, a Notch signaling pathway downstream gene. RT-qPCR substantiated these results and this was accompanied by a concomitant decrease in stemness markers Oct4, Sox2, and Nanog up to day 16. Our flow cytometric data show that during the differentiation of the ad-MSCs cell cycle-associated genes were downregulated and the population doubling time was increased. This is typical of cells undergoing differentiation. Sox9, *p*-TGFβRII, and Notch1 are known to be involved in the regulation of chondrogenesis and in preserving the chondrocyte phenotype [44,45,46,47]. Sox9 is well established as a master regulator of chondrogenesis and therefore our results are in agreement with the specific role for Sox9 in regulating chondrogenesis [46,47]. Sox9 is known to regulate chondrogenesis via the activation of Sox5 and Sox6, while Notch signaling has been identified as a regulator of Sox9 in chondrocytes [44,46]. It has been established that Notch signaling is necessary for normal onset of chondrocyte maturation [44,45,46,47]. Our data are in agreement with Notch1 being upstream of Sox9 and therefore regulating it. Notch signaling is known to influence proliferation [48,49] and we observed a decrease in proliferation of ad-MSCs in this study in the presence of fd-ECM.

Long-term culture and passaging of cells have been shown to cause senescence of cells including MSCs [41,42]. These studies show that long-term culture of MSCs results in changes such as telomere shortening [41,42,43]. If this occurs during therapy it can result in reduced therapeutic efficacy [50,51,52]. Therefore we evaluated the influence of the ECM on the passaging of ad-MSCs. Based on senescence-associated gene expression, fd-ECM prevented senescence. In fact, we observed that ad-MSCs cultured on fd-ECM exhibited an increased expression of genes such as *hTERT* and *bFGF* and a decrease in the expression of genes such as *P16* and *P21*. *hTERT* is known to play a significant part in the shortening of telomere during cellular senescence. These results demonstrate the fd-ECM does not cause senescence during ad-MSC culture. Reactive oxygen species can cause DNA damage to cells and this can lead to induction of senescence genes such as *P53* and *P21*. Our results also show that the fd-ECM reduces the H_2_O_2_-mediated increase in cleaved caspase 3 and 9. In vitro culture of MSCs, under certain conditions such as stress and hypoxia, has been suggested to cause spontaneous cellular transformation [53], although this is not a commonly reported finding. Our analysis of specific transformation markers after culture of the MSCs for up to 24 days on fd-ECM shows that most genes involved in tumor activation, suppression and DNA repair remain unchanged in the control versus fd-ECM. In fact, the expression of genes such as *P21* and *P53* was reduced when ad-MSCs were cultured on fd-ECM.

It is possible that fd-ECM can be used as a patch containing ad-MSCs and, based on our results, the fd-ECM will induce chondrogenic differentiation of the ad-MSCs. However, this might not be the case in vivo and the use of the patch might result in an immune reaction. Further experiments are therefore necessary to determine the usability of the fd-ECM as a patch and also to generate chondrogenically differentiated ad-MSCs for clinical use.

## 4. Materials and Methods

### 4.1. Reagents

Most reagents were sourced from GIBCO BRL Life Technologies (Gaithersburg, MD, USA), BioRad (Foster City, CA, USA) and Merck Biosciences (Darmstadt, Germany). Professor Igor Prudovsky (Maine Medical Center Research Institute, Scarborough, ME, USA) kindly provided the dominant negative mutant Notch1 expression plasmid.

### 4.2. Preparation of Fd-ECM and Cell Culture

WI-38 fibroblasts were used in the preparation of the fd-ECM as described before [5,6,20]. The WI38 cell line was sourced from American Type Culture Collection (ATCC, Manassas, VA, USA). Briefly, WI38 cells were grown in Dulbecco’s Modified Eagle’s Media (DMEM) containing 10% heat inactivated fetal bovine serum (FBS) (GIBCO, New York, NY, USA), 2 mM l-glutamine (GIBCO), 100 U/mL penicillin (Biochrom, Berlin, Germany), and 100 µg/mL streptomycin (Biochrom). Every alternate day there was the addition of ascorbic acid (50 μg/mL) (Sigma Aldrich, St. Louis, MO, USA). Confluent cells were grown for a further eight days and lysed through the addition of 20 mM ammonium hydroxide (Sigma Aldrich, Steinheim, Germany) for 1 min. Sterile phosphate-buffered saline (PBS) was used to wash the fd-ECM three times. The ECM was then air dried. The fd-ECM was used immediately or stored at 4 °C to maintain the protein integrity [5,20,54,55]. The fd-ECM was washed three times with sterile PBS before use.

### 4.3. Adipose Tissue Procurement and Processing

Adipose tissue was sourced from patients undergoing liposuction procedures. Informed consent was obtained from all volunteers prior to sample collection according to institutional guidelines. All procedures were done according to the Declaration of Helsinki guidelines. Approval was obtained before commencement of the study from the Research Ethics Committee at the Faculty of Healthy Sciences, University of Pretoria, South Africa (Pretoria, South Africa; Register I.D.: FWA 00002567; IRB 00002235 IORG0001762; Protocol Number: 218/2010). Mesenchymal stromal/stem cells isolation was performed as previously described [11,13,14]. Prior to tissue digestion with 0.001 mg/mL Type I collagenase (GIBCO, Grand Island, NY, USA), PBS was used to wash the adipose tissue and it was centrifuged twice for 3 min at 1152× *g* in sterile PBS supplemented with penicillin and streptomycin. The pellets were collected as ad-MSCs and cultured in α-minimal essential medium (α-MEM) containing 10% FBS (*v*/*v*) (GIBCO, Life Technologies, New York, NY, USA), 50 U/mL penicillin, and 50 μg/mL streptomycin and cultured at 37 °C. Culture dishes (100 mm culture dish) were used and 5 × 10^5^ ad-MSCs were cultured. Cells were cultured overnight and then washed with PBS supplemented with penicillin and streptomycin to remove non-adherent cells. Cells at 80% confluency were harvested using Trypsin-EDTA and designated as passage 0. Media were changed every 2–3 days. For the experiments described in this manuscript, ad-MSCs preparations were used at passages 6–10.

### 4.4. Immunophenotyping and Differentiation of Ad-MSCs

In order to analyze surface marker expression profiles of mesenchymal stromal/stem cells, flow cytometric analysis was performed using mouse anti-human fluorochrome-conjugated monoclonal antibodies. Several monoclonal antibodies were sourced from Beckman Coulter (Miami, FL, USA), BioLegend (San Diego, CA, USA), and eBioscience (San Diego, CA, USA) and used in the characterization of the ad-MSCs: CD34-PC7/-ECD/-PE/-FITC, CD45-PC5/-PC7/-ECD, CD73-APC/-FITC, CD90-FITC/-PC5, and CD105-PE. For each sample, a 100-µL cell aliquot was incubated at 37 °C for 15 min in the dark after the addition of a panel of monoclonal antibodies. Following incubation, cells were washed three times with PBS supplemented with 2% FBS, re-suspended in PBS, and analyzed for antigen expression. Results were obtained using a FC500 MCL (5 colors, 1 laser configuration) and Gallios (10 colors, 3 lasers configuration) flow cytometer (from Beckman Coulter). A minimum of 5 × 10^3^ intact cells were analyzed during data acquisition. The Kaluza Flow Cytometry analysis software 1.2 was used to analyze the data (Beckman Coulter). For adipogenic, osteogenic, and chondrogenic differentiation, ad-MSCs were incubated with the respective differentiation media for up to 21 days. Change of media was done twice every week. Once the cells were 70%–80% confluent, they were induced to differentiate. Adipogenic-inducing medium consisted of Dulbecco’s Modified Eagle’s Medium (DMEM 1× + GlutaMAX™, GIBCO by Life Technologies™, Grand Island, NY, USA) containing 10% FBS, 1% 1 μM dexamethasone (Sigma-Aldrich Chemie, Steinheim, Germany), 0.5 mM 3-isobutyl-methylxanthine (Sigma-Aldrich Chemie), 200 μM indomethacin (Sigma-Aldrich Chemie), and 10 μg/mL insulin (human recombinant Zinc, GIBCO by Life Technologies™). After 21 days of differentiation, 4% formaldehyde (Sigma-Aldrich Chemie) solution (Sigma-Aldrich Chemie) was used to fix the cells (60 min) and they were stored at 4 °C in PBS. Cells were stained with 0.3% Oil Red O (ORO, Sigma-Aldrich Chemie) solution and counter stained with 1 mL 0.01% Toluidine Blue O (TBO containing 0.01% Na_2_CO_3_ (Sigma-Aldrich Chemie)) for 5 min to detect accumulation of lipid droplets. Images were acquired using a fluorescence microscope (Zeiss Axiovert 200, Carl Zeiss Werke, Göttingen, Germany). Osteogenic-inducing medium consisted of DMEM supplemented with 10% FBS, 0.1 μM dexamethasone, 50 μM ascorbate-2-phosphate (Sigma-Aldrich Chemie), and 10 mM β-glycerophosphate (Sigma-Aldrich Chemie). After 21 days of differentiation, the cells were fixed using 4% formaldehyde solution (60 min) and stored at 4 °C in PBS. Mineralization was detected by staining the cells with 2% Alizarin Red S (ARS, Sigma-Aldrich Chemie). Images were acquired using a fluorescence microscope. The “pellet culture system” was used for chondrogenic differentiation with minor modifications. A chondrogenic-inducing medium consisting of DMEM containing 0.1 μM dexamethasone, 50 µg/mL ascorbate-2-phosphate, 10 ng/mL transformed growth factor β-3 (TGF-β3, GIBCO by Invitrogen™), 40 µg/mL proline (Merck, Darmstadt, Germany), 100 µg/mL pyruvate (Merck), and 1% insulin, human transferrin and selenous acid (ITS™) premix universal culture supplement (BD Biosciences, Bedford, OH, USA) was used for 21 days. After 21 days, the pellets were fixed in 4% formaldehyde and stored in PBS until further processing. Pellets were removed from the PBS and serially dehydrated (for 15 min per ethanol change) in 30%, 50%, 70%, and 90% ethanol, followed by dehydration in absolute ethanol. The sample was then infiltrated with a 50% London Resin (LR) white medium-grade acrylic resin (SPI supplies, West Chester, PA, USA) in absolute ethanol solution for one hour, followed by infiltration with 100% LR White Resin for a minimum of four hours. Sections were collected onto droplets of water on glass slides and dried on a slide warmer, stained with 1% TBO (1% Na_2_CO_3_), and images captured with a fluorescence microscope using a 10× magnification objective lens to confirm chondrogenic differentiation. Positive staining of the proteoglycans (purple) was an indication of chondrogenic differentiation.

### 4.5. RNA Preparation and RT-qPCR

Total ribonucleic acid (RNA) was extracted from ad-MSCs as described before [5,6,56]. Complementary deoxyribonucleic acid (cDNA), generated from 5 μg of total RNA, was used in the real-time quantitative polymerase chain reactions (RT-qPCR). RT-qPCR was performed and monitored using the Light Cycler 480 II (Roche, Mannheim, Germany). Samples were analyzed using primers listed in Table S1 (all: Whitehead Scientific, Cape Town, South Africa). Thermocycling for all targets was carried out the following conditions: initial denaturation at 94 °C for 5 min followed by 35 cycles of 94 °C for 20 s, 55 °C for 20 s, and 72 °C for 20 s. Genes analyzed along with their respective primer sequences are given in Table S1. Expression levels of osteogenic markers (CBFA1, OC), chondrogenic markers (COL2A1, Sox9), and adipogenic markers (peroxisomal proliferator-activated receptor γ 2 (PPARg2) and LPL) were analyzed by RT-qPCR. RT-qPCR experiments were done in triplicate and significant differences are shown by * *p* < 0.05.

### 4.6. Cellular Adhesion Assays

Ad-MSCs were seeded onto plastic and fd-ECM-coated six-well cell culture plates and allowed to attach for 2 h at 37 °C. Cells that were not attached were then washed off with PBS, leaving attached cells on the plastic dish surface or on the fd-ECM. Adherent cells were incubated with 0.5 mg/mL MTT solution 3-(4,5-dimethylthiazol-2-yl)-2,5-diphenyltetrazolium bromide (Promega, Madison, WI, USA) for 2 h at 37 °C. PBS was used to wash the cells and 0.30 mL acidic isopropanol was added. Plates were incubated to dissolve the converted dye. Absorbance was then measured at 570 nm on an enzyme-linked immunosorbent assay reader. The relative attachment potential of ad-MSCs on fd-ECM was compared to controls.

### 4.7. Ad-MSCs Doubling Time (Gt)

Ad-MSCs doubling time (*PD*) is the time it takes the population of ad-MSCs to double and was calculated based on the following formula:

*PD* = *t* × ln2/ln(*FCC*/*SCC*)

where *t* equals time in h, ln represents the natural logarithm, *FCC* represents the final ad-MSC cell number, and *SCC* represents the starting ad-MSC cell number.

### 4.8. Immunoblot Analysis

Immunoblot analysis was done as described before [10]. Ad-MSCs were washed twice with PBS, lysed in radioimmunoprecipitation assay (RIPA) buffer, and a protease inhibitors mixture was added. The protein concentration of the resulting lysates was determined using the bicinchoninic (BCA) assay. Proteins (50 μg) were electrophoresed on 10% SDS-PAGE gels in the presence of 50 mM β-mercaptoethanol. Transfer of proteins was done using a nitrocellulose membrane. Fat-free milk (5%, *w*/*v*) in Tris Buffered Saline (TBS) containing Tween-20 was used to block the membranes. Overnight incubation of the membranes was done at 4 °C with different primary antibodies: anti-Sox9, anti-Oct4, anti-CD44, anti-PCNA, anti-Nanog, anti-Gata 3, anti-Notch1, anti-Runx 2 (all from Cell Signaling Technology, Beverly, MA, USA), anti-cleaved caspase 3, anti-cleaved caspase 9, anti-caspase 3, anti-caspase 9, anti-β-catenin, anti-VEGF, anti-bFGF, anti-p21, anti-p27, anti-p53, anti-vimentin, ant-COL1A1, anti-COL II, anti-Ki67, anti-cyclin D1, anti-cyclin B1, anti-cyclin A, anti-*p*-β-catenin, anti-Osteopontin, anti-*p*-TGFβRII, anti-Jagged1, and anti-GAPDH (Santa Cruz, Biotechnology, Santa Cruz, CA, USA). The membranes were washed twice with TBS-T and incubation was done with secondary antibodies conjugated to horseradish peroxidase-conjugated (HRP) (BioRad). Detection was then done using Lumiglo substrate (KPL, Gaithersburg, MD, USA). All experiments were repeated three times.

### 4.9. Cell Cycle Analysis

Approximately 5 × 10^4^ ad-MSCs were cultured on control dishes and on fd-ECM for different incubation times up to a maximum of passage 16. For longer incubation times, passaging was done every four days. The cells were detached from the control dishes and the fd-ECM, and processed for flow cytometry analysis. Control and fd-ECM cultured ad-MSCs were washed with PBS and fixed in 70% ethanol for 1 h at 4 °C. Ad-MSCs were then stained with propidium iodide (50 µg/mL propidium iodide) and RNase A (10 µg/mL) was added for 3 h at 4 °C. Analysis was then done using a FACScan cell sorter (Becton Dickinson, Franklin Lakes, NJ, USA). Approximately 1 × 10^4^ cells were analyzed. Cellquest software (Version 5.1, Becton Dickinson, Franklin Lakes, NJ, USA) was used to determine the cell cycle profiles.

### 4.10. Transient Transfection Assay

The Transfectin reagent (BioRad, Munich, Germany) was used in the transient transfection assays. Ad-MSCs were cultured on plastic or on the fd-ECM. The media was then changed and the Notch1 siRNA, 2× β-catenin siRNA, dominant negative mutant Notch1 construct (dnNotch1), and control vectors were added to the cells. Incubation was continued for up to two days, after which cell lysates were obtained. RNA was extracted and used in RT-qPCR analysis. Immunoblot analysis was then performed to measure various proteins levels.

### 4.11. Statistical Analysis

GraphPad Prism (Version 5, GraphPad Software Inc, La Jolla, CA, USA) was used to perform the statistical analysis. All data are presented as means ± standard deviation (S.D.). Statistical significance was evaluated using the paired Student’s *t* test. Statistical significance is shown by * *p <* 0.05.

## 5. Conclusions

This study extends our understanding of how ad-MSCs behave and function in the context of a cellular niche provided by fd-ECM. How ad-MSCs differentiate when placed in different environments is vital prior to their use in tissue engineering and regenerative medicine. To the best of our knowledge, this study is the first study to evaluate the influence of an fd-ECM on ad-MSCs’ growth kinetics and differentiation. Based on our results, the fd-ECM directs ad-MSC differentiation along the chondrogenic lineage. We should mention that further analysis on the precise signaling pathways implicated in this process must still be elucidated. Further in vitro and in vivo experiments must be done to determine the long-term effect of fd-ECM use before it can be used in stem cell therapy procedures. With cartilage defects being one of the main problems experienced by many people, chondrogenically differentiated ad-MSCs represent a viable therapeutic option for the treatment of such defects.

## Supplementary Materials

Supplementary materials can be found at http://www.mdpi.com/1422-0067/17/8/1259/s1.
