# Supplementary material for: Fibroblast-Derived Extracellular Matrix Induces Chondrogenic Differentiation in Human Adipose-Derived Mesenchymal Stromal/Stem Cells in Vitro
Source: Int J Mol Sci. 2016 Aug 3;17(8):1259. doi: 10.3390/ijms17081259 (PMC5000657; doi:10.3390/ijms17081259)
Supplement: Supplementary file 1 [file ijms-17-01259-s001.pdf]

Supplementary Materials: Fibroblast-Derived Extracellular Matrix Induces Chondrogenic Differentiation in Human Adipose-Derived Mesenchymal Stromal/Stem Cells in Vitro

Kevin Dzobo, Taegyn Turnley, Andrew Wishart, Arielle Rowe, Karlien Kallmeyer, Fiona A. van Vollenstee, Nicholas E. Thomford, Collet Dandara, Denis Chopera, Michael S. Pepper and M. Iqbal Parker

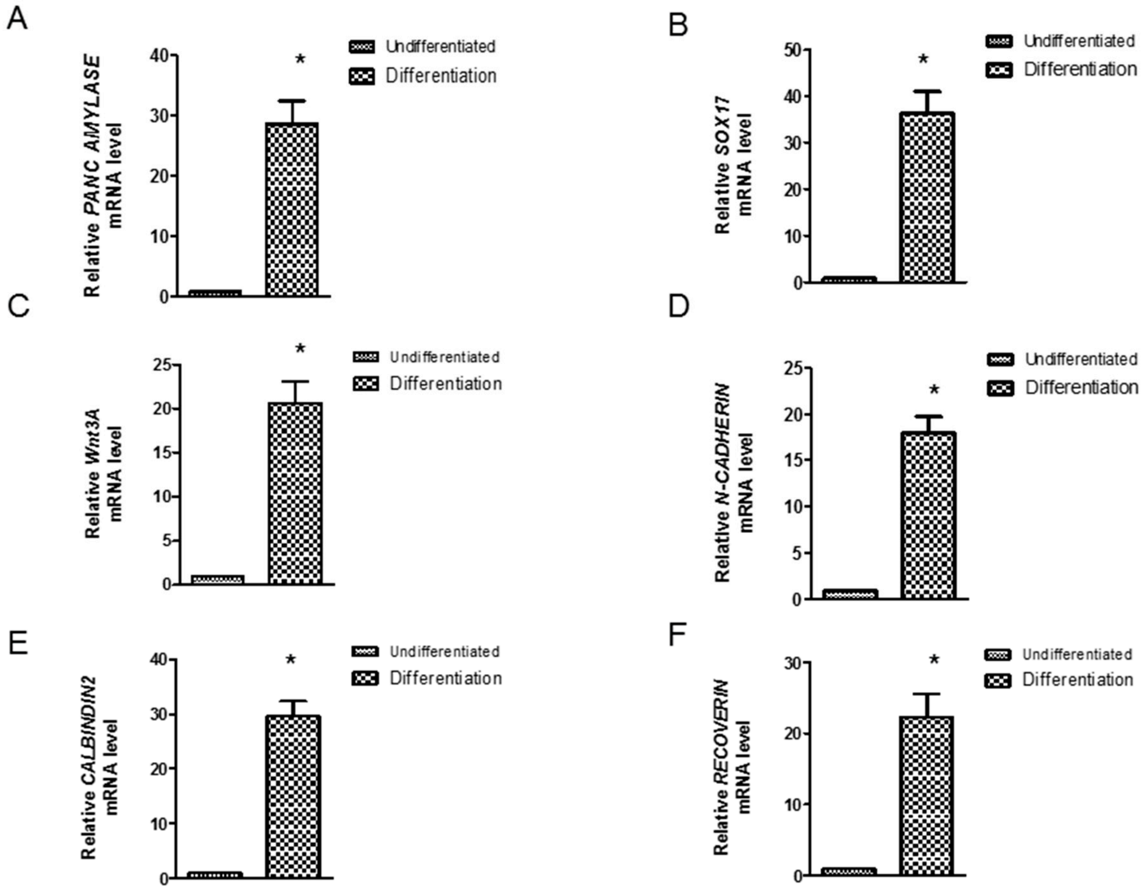

Figure S1. Induced differentiation capacity of ad-MSCs. Lineage-specific differentiation capacity of ad-MSCs. Ad-MSCs were cultured for 21 days in the respective differentiation media and then RT-qPCR was done to evaluate lineage-specific markers. (**A**,**B**) Endodermal differentiation was evaluated by RT-qPCR analysis for *PANCREATIC AMYLASE* mRNA and *SOX17* mRNA; (**C**,**D**) Mesodermal differentiation was evaluated by RT-qPCR analysis for Wnt Family Member 3a: *WNT3A*, and *N-CADHERIN* mRNA; and (**E**,**F**) Ectodermal differentiation was evaluated by RT-qPCR analysis of *CALBINDIN2* and *RECOVERIN* mRNA. * $p < 0.05$.

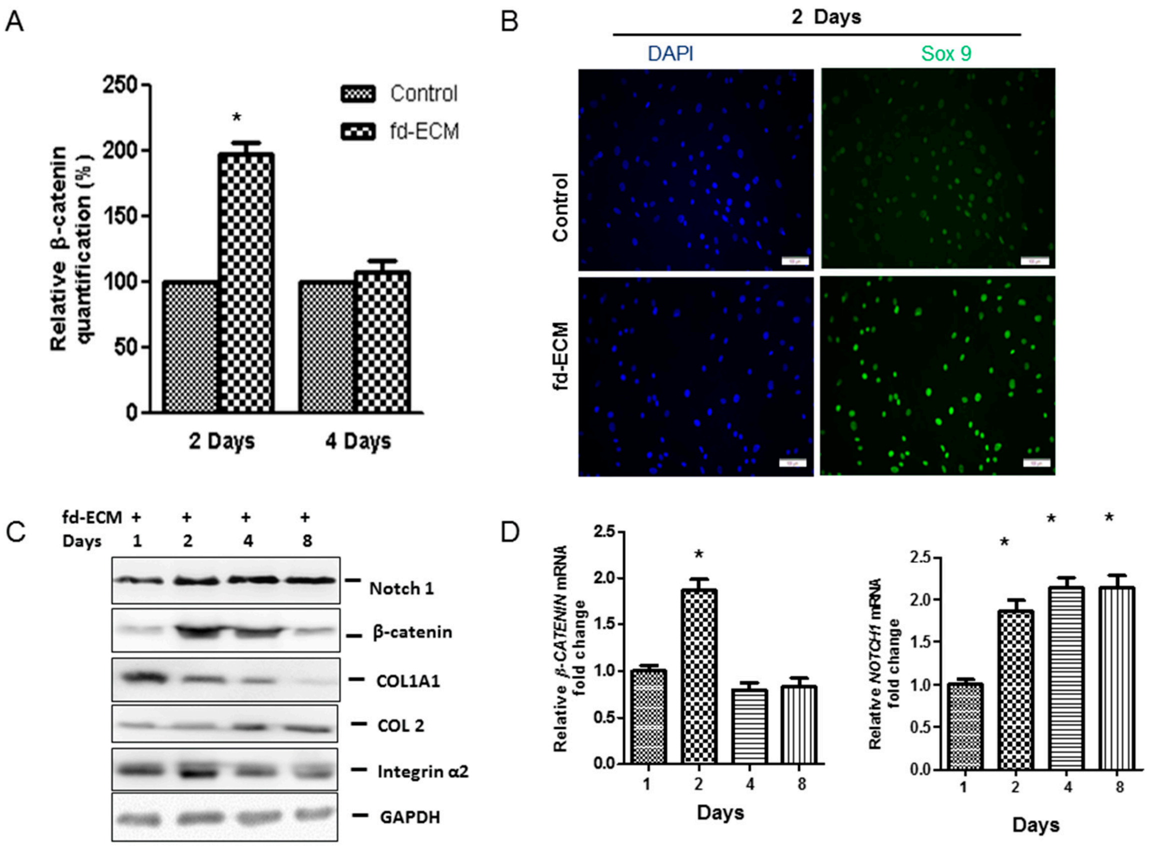

Figure S2. β-Catenin, Sox9 and Notch1 signaling are involved in fd-ECM-mediated chondrogenic differentiation of ad-MSCs. * $p < 0.05$. (**A**) Densitometric quantification of β-catenin levels as shown in Figure 8A,B; (**B**) Ad-MSCs cultured on control plastic dishes and on fd-ECM were evaluated for Sox 9 expression using immunofluorescence assay. Scale bar: 100 μm; (**C**) Ad-MSCs were cultured on fd-ECM (+) for the indicated time periods and incubated with antibodies against Notch1, β-catenin, COL1A1, type II collagen and integrin *α*2; and (**D**) Ad-MSCs were cultured on fd-ECM for the indicated periods and RT-qPCR was done to evaluate the expression of *β-CATENIN* and *NOTCH1.*

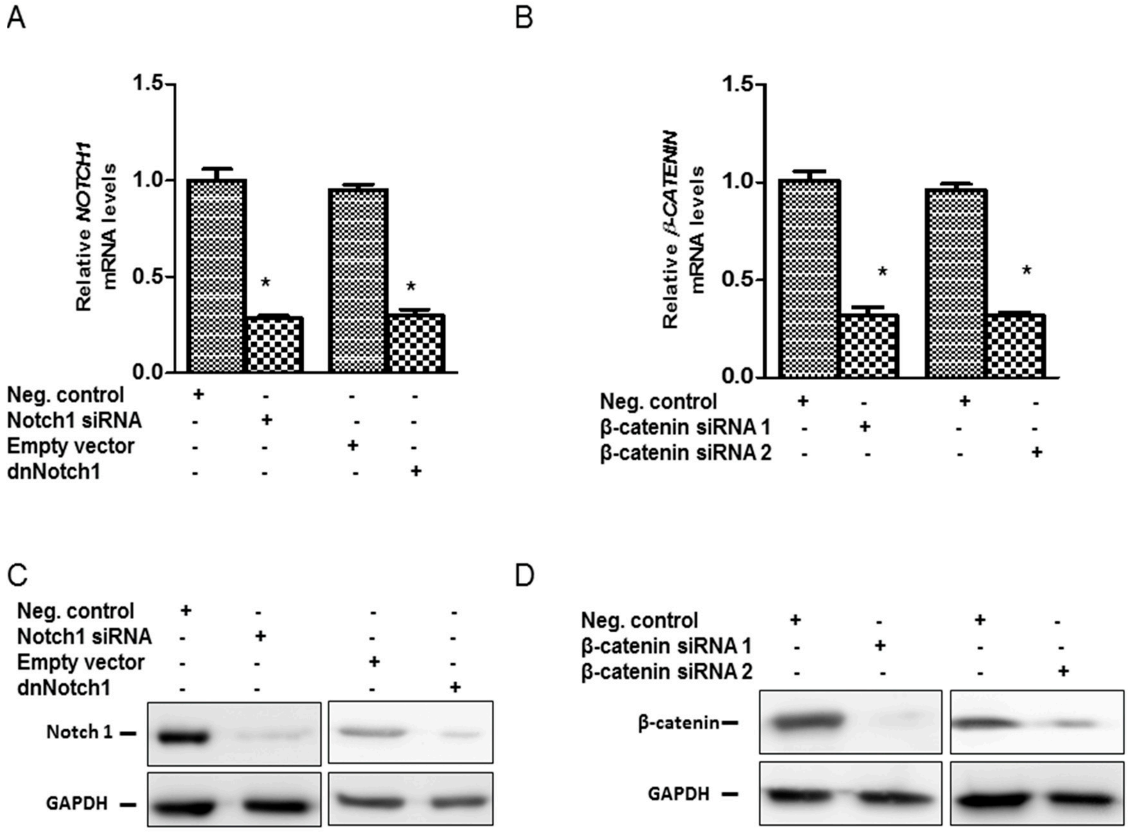

Figure S3. Knockdown of Notch1 and β-catenin mRNA and protein using siRNA (+) and a dominant negative mutant Notch1 construct (+) or the ad-MSCs were transfected with control siRNA (−). (**A**) Ad-MSCs were transfected with Notch1 siRNA (+) and a dominant negative mutant Notch1 construct (+) and *NOTCH1* mRNA levels were evaluated; (**B**) Ad-MSCs were transfected with two β-catenin siRNA (+) and RT-qPCR was performed to evaluate *β-CATENIN* mRNA levels; (**C**) Immunoblot analysis of Notch1 protein levels after Ad-MSCs were transfected with Notch1 siRNA (+) and dnNocth1 (+); and (**D**) Immunoblot analysis of β-catenin protein levels after Ad-MSCs were transfected with two β-catenin siRNA (+). * $p < 0.05$.

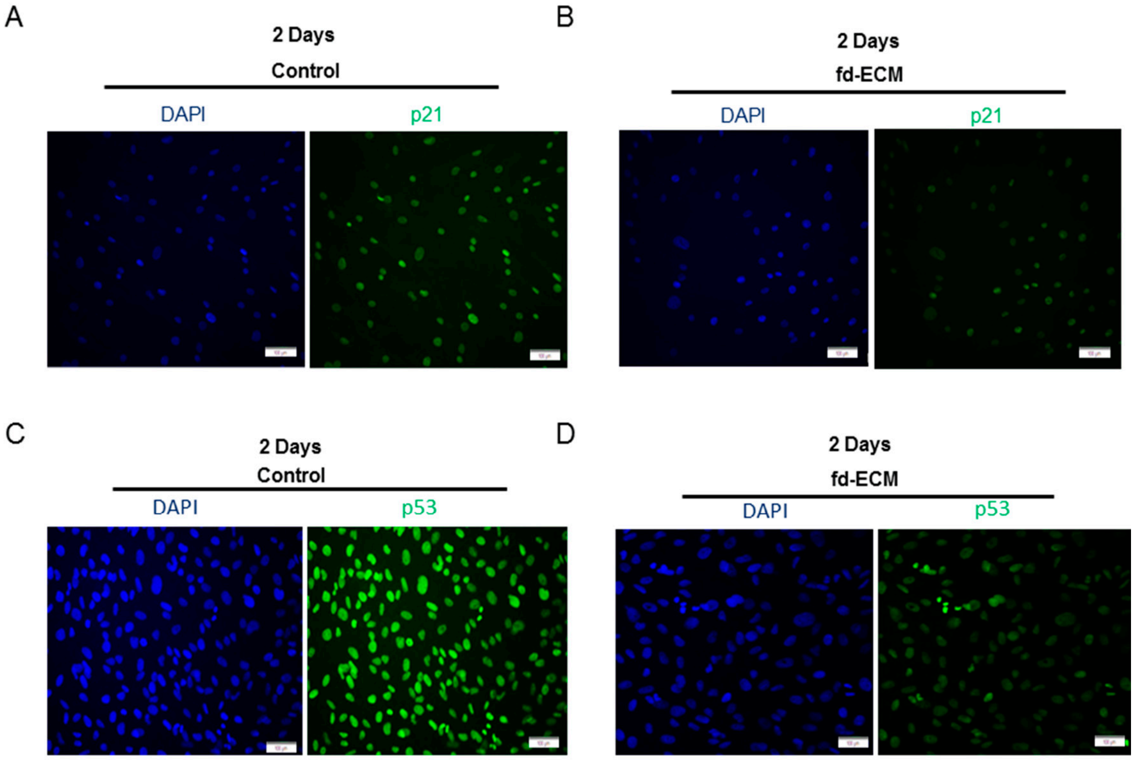

Figure S4. Fd-ECM downregulates p21 and p53 gene expression in ad-MSCs. (**A**) Immunofluorescence analysis of p21 expression in Ad-MSCs cultured on control plastic dishes for 2 days; (**B**) Immunofluorescence analysis of p21 expression in Ad-MSCs cultured on fd-ECM for 2 days; (**C**) Immunofluorescence analysis of p53 expression in Ad-MSCs cultured on control plastic dishes for 2 days; and (**D**) Immunofluorescence analysis of p53 in Ad-MSCs cultured on fd-ECM for 2 days. Scale bar: 100 μm.

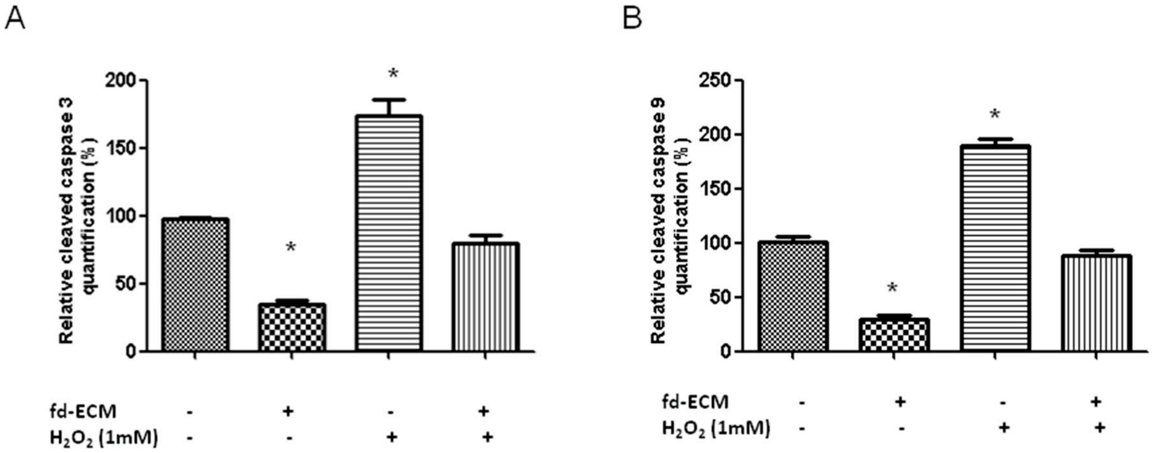

Figure S5. Densitometric quantification of cleaved caspases 3 (**A**) and 9 (**B**), as shown in Figure 9D in the presence of the fd-ECM (+) or on control plastic dishes (−). * $p < 0.05$.

Table S1. Oligonucleotide primer sequences used for RT-qPCR.

Gene	Forward	Reverse
GAPDH	5′-GCTCTCCAGAACATCATCC-3′	5′-GCCTGCTTCACCACCTTC-3′
c-MYC	5′-AAGACTCCAGCGCCTTCTCTC-3′	5′-GTTTTCCAACTCCGGGATCTG-3′
PCNA	5′-CCATCCTCAAGAAGGTGTTGG-3′	5′-GTGTCCCATATCCGCAATTTTAT-3′
CYCLIN D1	5′-ACAAACAGATCATCCGCAAACAC-3′	5′-CTTGGACTCCTCGGGGTTGT-3′
P21	5′-AGGGGACGGTCATCTACAACC-3′	5′-ATGGCCTTGCCATAGGCTGAG-3′
P53	5′-TTGGATCCATGTTTTGCCAACTGGCC-3′	5′-TTGAATTCAGGCTCCCCTTTCTTGCG-3′
β-CATENIN	5′-GCTCTGGAGTTCTCTCATCG-3′	5′-GGTGGAAGGACTTAGGTTTG-3′
P16	5′-TTATTTGAGCTTTGGTTCTG-3′	5′-CCGGCTTTCGTAGTTTTCAT-3′
CBFA1	5′-GGTTCCAGCAGGTAGCTGAG-3′	5′-AGACACCAAACTCCACAGCC-3′
OC	5′-GACTGTGACGAGTTGGCTGA-3′	5′-GGAAGAGGAAAGAAGGGTGC-3′
COL2A1	5′-CCTCTGCGACGACATAATCT-3′	5′-CTCCTTTCTGTCCCTTTGGT-3′
SOX9	5′-TAAAGGCAACTCGTACCCAA-3′	5′-ATTCTCCATCATCCTCCACG-3′
PPARG2	5′-ATGACAGCGACTTGGCAATA-3′	5′-GGCTTGTAGCAGGTTGTCTT-3′
NOTCH1	5′-GACATCACGGATCATATGGA-3′	5′-CTCGCATTGACCATTCAAAC-3′
RUNX 2	5′-AGTAGCCAGGTTCAACGAT-3′	5′-GGAGGATTTGTGAAGACTGTT-3′
GATA3	5'-TGTCTGCAGCCAGGAGAGC-3′	5'–ATGCATCAAACAACTGTGGCCA-3'
OSTEOPONTIN	5′-AGTTTCGCAGACCTGACATCCAGT-3′	5′-TTCATAACTGTCCTTCCCACGGCT-3′
TNF-α	5′-CCCAGGGACCTCTCTCTAATC-3′	5′-ATGGGCTACAGGCTTGTCACT-3′
LPL	5′-AGGAGCATTACCCAGTGTCC-3′	5′-GGCTGTATCCCGGGAGATGGA-3′
OCT4	5′-GCCCGAAAGAGAAAGCGAAC-3′	5′-AACCACACTCGGACCACATC-3′
SOX2	5′-CCCAGCAGACTTCACATGT-3′	5′- CCTCCCATTTCCCTCGTTTT-3′
NANOG	5′-GCTTGCCTTGCTTTGAAGCA-3′	5′-TTCTTGACTGGGACCTTGTC-3′
HES1	5′- CCAGTTTGCTTTCCTCATTCC-3′	5′-TCTTCTCTCCCAGTATTCAAGTTCC-3′
PANC AMYLASE	5′-AGTTAGCTATAAATTATGCACAAG-3′	5′-TGCTTGGAAGCATCAAGTCTGAAC-3′
SOX17	5′-CGCACGGAATTTGAACAGTA-3′	5′-GGATCAGGGACCTGTCACAC-3′
WNT3A	5′-TGTTGGGCCACAGTATTCCT-3′	5′-ATGAGCGTGTCACTGCAAAG-3′
N-CADHERIN	5'-GGTGGAGGAGAAGAAGACCAG-3'	5'-GGCATCAGGCTCCACAGT-3'
CALBINDIN2	5′-GCGGCTACATTGACGAGCAT-3′	5′-GCAGCAGAAGCAGGGTTTGG-3′
RECOVERIN	5′-GGAAAAGCGAGCCGAGAAGA-3′	5′-CCTGGGGTGGATGTGTGTGT-3′
RAD51	5′-TTTGGAGAATTCCGAACTGG-3′	5′-AGGAAGACAGGGAGAGTCG-3′
ERCC3	5′-CCAGGAAGCGGCACTATGAGG-3′	5′-GGTCGTCCTTCAGCGGCATTT-3′
XRCC4	5′-AAGATGTCTCATTCAGACTTG-3′	5′-CCGCTTATAAAGATCAGTCTC-3′

GAPDH: Glyceraldehyde 3-phosphate dehydrogenase; PCNA: Proliferating cell nuclear antigen; CBFA1: Core-binding factor subunit α1; OC: Osteocalcin; COL2A1: Collagen type 2; SOX9: Sex determining region Y-box 9; SOX2: Sex determining region Y-box 2; PPARG2: Peroxisome proliferator-activated receptor γ 2; LPL: Lipoprotein lipase; Runx2: Runt-related transcription factor 2; Gata3: GATA Binding Protein 3; TNF-α: Tumor necrosis factor α; OCT4: Octamer-binding transcription factor 4; Hes1: Hairy and enhancer of split-1; Panc amylase: Pancreatic amylase: Sox17: Sex determining region Y-box 17; Wnt3a: Wnt Family Member 3a; ERCC3: Excision Repair Cross-Complementation Group 3; XRCC4: X-ray repair cross-complementing protein 4.
